# Differential Diagnostic Models Between Vasovagal Syncope and Psychogenic Pseudosyncope in Children

**DOI:** 10.3389/fneur.2019.01392

**Published:** 2020-01-23

**Authors:** Zhening Zhang, Xingyuan Jiang, Lu Han, Selena Chen, Ling Tao, Chunyan Tao, Hong Tian, Junbao Du

**Affiliations:** ^1^Department of Pediatrics, Peking University First Hospital, Beijing, China; ^2^Research Unit of Clinical Diagnosis and Treatment of Pediatric Syncope and Cardiovascular Diseases, Chinese Academy of Medical Sciences, Beijing, China; ^3^Department of Pediatric Cardiology, Children's Hospital of Fudan University, Shanghai, China; ^4^Division of Biological Sciences, University of California, San Diego, San Diego, CA, United States; ^5^Key Laboratory of Molecular Cardiovascular Sciences, Ministry of Education, Beijing, China

**Keywords:** vasovagal syncope, psychogenic pseudosyncope, differential diagnosis, scoring model, logistic regression model

## Abstract

**Objective:** We aimed to establish useful models for the clinical differential diagnosis between vasovagal syncope (VVS) and psychogenic pseudosyncope (PPS).

**Methods:** This bicentric study included 176 patients (150 VVS and 26 PPS cases) for model development. Based on the results of univariate and multivariate analyses, a logistic regression model and a scoring model were established and their abilities to differentiate VVS from PPS were tested. Another 78 patients (53 VVS and 25 PPS) were used for external validation.

**Results:** In the logistic regression model, the outcome indicated that the QT-dispersion (QTd) (*P* < 0.001), syncope duration (*P* < 0.001), and upright posture (*P* < 0.001) acted as independent factors for the differentiation of VVS from PPS, which generated an area under the curve (AUC) of 0.892. A cutoff value of 0.234 yielded a sensitivity and specificity of 89.3 and 80.8%, respectively, for the differentiation between VVS and PPS in the logistic regression model. In the scoring model which consists of three variables, a cutoff score of three points yielded a sensitivity and specificity of 91.3 and 76.9%, respectively, with an AUC of 0.909. The external validation test indicated that the negative and positive predictive values of the scoring model were 78.8 and 91.7%, respectively, and the accuracy was 80.8%.

**Conclusion:** The scoring model consisting of three variables is an easy-to-perform, inexpensive, and non-invasive measure for initial differential diagnosis between VVS and PPS.

## Introduction

Syncope is categorized as a transient loss of consciousness (TLOC) associated with the incapacity to maintain posture (1). Vasovagal syncope (VVS), which accounts for 60–70% of the causes of syncope, is prevalent in children (2). Classified as a psychiatric disorder other than true TLOC, psychogenic pseudosyncope (PPS) shares several clinical similarities with VVS in clinical settings (3). Prodromal presyncope symptoms, such as changes in vision, shivering, sweating, and dyspnea, overlap in VVS and PPS patients (4, 5). Falling is often a manifestation of patients with VVS and PPS. Causative factors of VVS, including long-term upright posture, environmental stuffiness and emotional stress, are also occasionally found in PPS (6, 7). As a result, it is sometimes difficult for physicians to make an initial differential diagnosis between these two diseases in daily clinical practice.

It was previously found that the prolonged period of TLOC is more common in children with PPS than with VVS (8). However, there is insufficient evidence in support of this finding. Furthermore, direct observation of the clinical events is impractical in most situations, making it more difficult for physicians to distinguish VVS from PPS (7). It is worth noting that there are few targeted reports that quantitatively address the differential diagnosis between these two diseases. Although the head-up tilt test (HUTT) has been used as a valid measure for the differential diagnosis (9), its time consumption, unavailability in elementary hospitals and the risk of inducing critical complications including cardiac arrest and shock (10) limit its wide application across all clinical settings, especially in outpatient and elementary hospitals. As such, identifying an easy-to-perform, inexpensive. and non-invasive measure to initially differentiate VVS from PPS has long been an important issue in clinical research.

Therefore, the present study was undertaken to develop novel, feasible, and useful methods to help clinicians in the initial differential diagnosis between VVS and PPS in clinical practice.

## Methods

### Subjects

In the first part of this retrospective study, 482 children diagnosed with VVS and 52 children diagnosed with PPS were enrolled from the Department of Pediatrics at the Peking University First Hospital, China and Children's Hospital of Fudan University, China from January 2000 to January 2019. One hundred and fifty cases of VVS and 26 cases of PPS were ultimately included in our first part analysis according to the inclusion and exclusion criteria. We reviewed clinical records of those patients and used the data to develop a scoring model. In the second part of the study, we conducted an external validation test for the predictive values of our models. Another 53 children diagnosed with VVS and 25 children diagnosed with PPS from the Children's Hospital of Fudan University, China were enrolled and analyzed. This study was approved by the ethics committee of the local institution (Ethics Committee Number: 2018-202).

### Inclusion and Exclusion Criteria

The diagnostic criteria of VVS (11, 12) are: (1) presented with a history of syncope, accompanied by a subsequent spontaneous recovery; (2) usually induced by prolonged standing, emotional stress, or medical setting; (3) with prodromal features including diaphoresis, fever, flushing, nausea, visual blurring, or pale complexion; (4) with hypotension and/or inappropriate bradycardia during onset; (5) with positive HUTT results; (6) exclusion of other diseases including cardiovascular, neurogenic, or metabolic diseases. These diagnostic criteria are compliant with the latest ACC Guideline.

PPS belongs to a conversion disorder, which is classified as a psychiatric disease. Considering that accurate diagnosis has not been explored in PPS, we established our inclusion criteria in reference to DSM-V and previous studies (13, 14). We included the patients diagnosed with PPS who met the following conditions: (1) presented with a history of recurrent apparent syncope without true unconsciousness, accompanied by subsequent spontaneous recovery; (2) attacks are usually featured with concomitant eye closure, loss of muscle tone, and immobility; (3) with normal blood pressure (BP) and heart rate (HR) during onset; (4) with negative response in HUTT; (5) exclusion of other diseases including cardiovascular, neurogenic, or metabolic diseases.

All enrolled patients underwent strict inspections to exclude other similar diseases. For all patients, ultrasonic cardiogram (UCG) and electrocardiogram (ECG) were used to rule out organic heart diseases (or syncope with a cardiogenic cause). EEG monitoring, transcranial Doppler ultrasound and/or CT scan were conducted and presented normal among enrolled PPS patients, to rule out neurogenic diseases such as epilepsy. Patients were also ruled out when clear causes of syncope (such as situational syncope, hypoglycemia, or carotid sinus syndrome) were present. Notably, since co-occurrence of VVS and PPS may be found in rare cases, patients with a mixed pattern of VVS and PPS (e.g., PPS with positive HUTT results) were denoted complicated cases and eliminated from our study.

### Electrocardiogram

A standard 12-lead ECG tracing at 25 mm/s paper speed and 10 mm/mV amplitude was performed on all patients. QTd was determined in all 12 leads and mean results were calculated from three consecutive cardiac cycles. QTd was generated by calculating the difference between the maximum and the minimum QT interval measured in each ECG lead from the onset of QRS complex to the end of T wave. All ECG measurements were performed manually by two independent investigators who were unaware of the clinical outcomes. When measurements were not identical, the mean of the values was calculated.

### Other Clinical Data

Baseline demographic data including gender, age, height, weight, body mass index (BMI), were recorded for all subjects. Medical history of clinical presentations including syncope duration, inducement factors, and family history were obtained from inpatient medical records. Inducement factors included long-term upright posture, a confined environment, emotional stress, etc. Syncope duration referred to the time length of loss of consciousness on average. We defined a positive family history of syncope as the existence of syncope among linear relatives within two generations. Systolic blood pressure (SBP) and diastolic blood pressure (DBP) were measured when the patients remained calm and in supine during HUTT. Changes of BP, HR, and clinical manifestations during HUTT were recorded (data not shown).

### Statistical Analysis

SPSS 25.0 and GraphPad Prism 7.0 were used for statistical analysis and curve plotting. For all analyses, statistical significances were assessed with a *P* value of 0.05.

We first performed univariate analysis to identify variables significantly differentiated between VVS and PPS patients. The Shapiro-Wilk test was used to assess the normality of distribution. A 2-independent *t*-test or Mann-Whitney U-test was performed to compare the continuous variables when they were normally or non-normally distributed, respectively. A chi-square test was performed to compare categorical variables between the two groups. To select independent factors for differential diagnosis, multivariate logistic regression models with forward selection were established by using baseline demographic variables (age, gender, BMI) plus variables generated by univariate analysis. A probability value of *P* < 0.05 was indicated for inclusion into the model. Results were expressed as an odds ratio (OR) with a 95% confidence interval (CI). Goodness of fit of the regression model was tested with the Hosmer-Lemeshow test. The efficiency of this model was assessed using the receiver operating characteristic curve (ROC). The AUC was then calculated and the optimal cutoff value was determined by the maximum of Youden index.

In order to enhance the feasibility of this differential diagnosis model in clinical practice, a scoring model was generated with approximations. Continuous variables were converted to dichotomous variables by selecting a cut-off point based on ROC curves and the specific values were then adjusted based on clinical convenience. We next performed multivariate logistic regression analysis and created a clinical prediction rule by using OR to determine the weight of each variable. Score points were assigned for each variable accordingly. The total sum of points for each individual was calculated, and their final scores were used for assessment.

## Results

**Figure 1:**
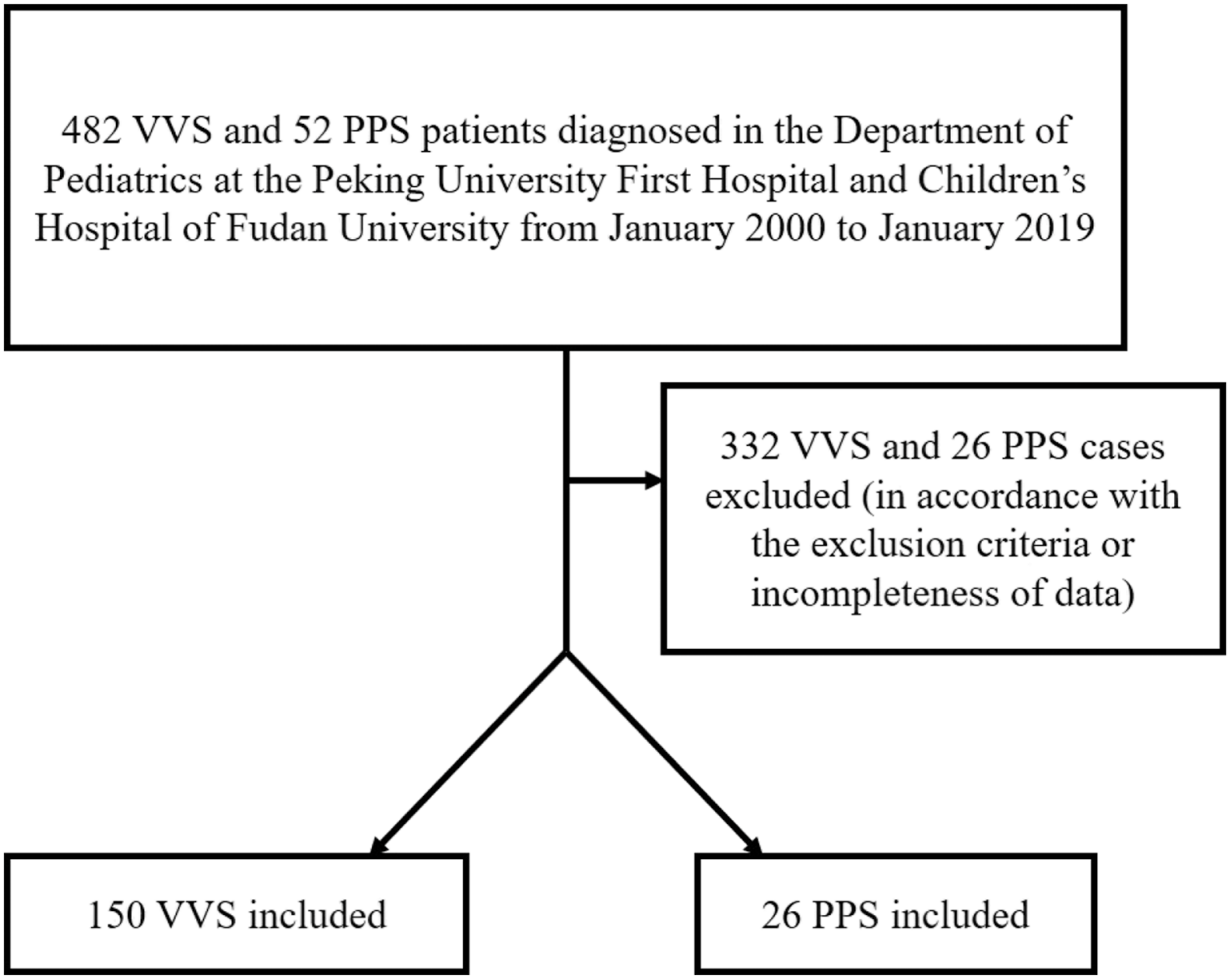
Flow chart of enrollment of study population in the first part of the study. PPS, psychogenic pseudosyncope; VVS, vasovagal syncope.

In the first part of the study, 150 VVS patients and 26 PPS patients were ultimately enrolled in this study (Figure 1). The VVS group contained 72 males and 78 females whose ages ranged from 5 to 17 years old. The PPS group contained 14 males and 12 females whose ages ranged from 7 to 15 years. Baseline demographic features of the patients are shown in Table S1.

There were significant differences between VVS and PPS patients in long-term upright posture (*P* < 0.001), syncope duration (*P* < 0.001), positive family history of syncope (*P* = 0.02), resting HR (*P* = 0.07), and QTd (*P* <0.001) as analyzed by univariate analysis (Table 1).

**Table 1:** Clinical data of patients diagnosed with vasovagal syncope (VVS) or psychogenic pseudosyncope (PPS).

**Group**	**Causative factors exposed (*****N*****)**			**Syncope duration (min)**	**FH of syncope (*N*)**	**Resting HR (bpm)**	**SBP (mmHg)**	**DBP (mmHg)**	**QTd (ms)**
	**Upright posture (Yes/No)**	**Stuffiness (Yes/No)**	**Emotional stress (Yes/No)**						
VVS	119/31	27/123	21/129	5.5 (2.5, 8.4)	36	77.1 (74.8, 79.3)	106.9 (105.1, 108.8)	62.3 (60.9, 63.8)	41.3 (38.7, 44.0)
PPS	5/21	3/23	4/22	50.0 (25.3, 74.8)	1	82.7 (77.0, 88.4)	102.3 (98.5, 106.1)	62.4 (59.2, 65.5)	27.9 (22.5, 33.3)
χ^2^/Z	38.454	0.277	0	−4.245	5.421	−1.817	−2.313	−0.359	−3.767
*P*	<0.001	0.60	1	<0.001	0.02	0.07	0.02	0.72	<0.001

*BMI, body mass index; DBP, diastolic blood pressure; FH, family history; HR, heart rate; PPS, psychogenic pseudosyncope; QTd, QT-dispersion; SBP, systolic blood pressure; VVS, vasovagal syncope*.

**Figure 2:**
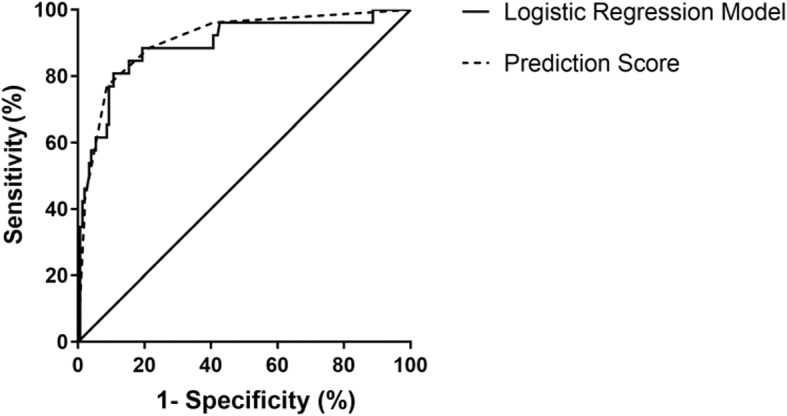
ROC curve of the diagnostic value of the logistic regression model and the scoring model between VVS and PPS. The y-axis represents sensitivity. The x-axis represents false positive rate (1-specificity). The 45° straight line stands for the reference line indicating sensitivity being equal to false positive rate. In the logistic regression model, the AUC was 0.892 (95% CI: 0.815–0.969; *P* < 0.001) and in the scoring model, the AUC of was 0.909 (95% CI: 0.845–0.973; *P* < 0.001). AUC, area under the curve; CI, confidence interval; PPS, psychogenic pseudosyncope; ROC, operation characteristic curve; VVS, vasovagal syncope.

We then performed binary logistic regression analysis and determined clinical factors as those with *P* < 0.05. Baseline data comprising age, gender, and BMI were set as independent variables, and the disease category was set as the dependent variable. We found that only upright posture (yes or no), syncopal duration (min), and QTd (ms) were independent predictors to differentiate PPS from VVS. The logistic regression equation went as: P = 1 / (1 + 1/ exp[0.022×D-2.117×U-0.049×QT + 0.565]), where “D” stood for the duration of a syncopal episode (min), “U” represented an upright position (1: with an upright position and 0: without), and “QT” represented QT-dispersion (ms). Predicting PPS with a *P* value > 0.234 and VVS with a *P* value < 0.234 yielded a sensitivity and a specificity of 89.3 and 80.8%, respectively, with an AUC of 0.892 (95% CI, 0.815–0.969; *P* < 0.001) in ROC analyses. Hosmer-Lemeshow statistics did not show any significance (*P* = 0.78). The scoring model was established subsequently. Based on ROC analysis for syncope duration and the feasibility of QTd in clinical application, we assigned the following scores according to the OR (Table 2): duration of a syncopal episode > 30 min: three points; without upright posture as an inducement: two points; and QTd < 31 ms: one point. A total score was calculated based on the sum of points for each patient. When the total points were ≥3, we predicted the likelihood of PPS, otherwise VVS. This scoring model indicated a sensitivity of 91.3% and a specificity of 76.9%, respectively. The AUC of the scoring model was 0.909 (95% CI: 0.845–0.973; *P* < 0.001). ROC curves of both models were plotted in one figure to show their agreement (Figure 2).

**Table 2:** Coefficients of binary logistic regression.

**Variables**	**Cut-off value**	**Variable assignments**	***p*-value**	**Odds ratio**	**Points**
Syncope duration	30min	“syncope duration > 30 min” = 1, “syncope duration ≤ 30” = 0	<0.001	14.794 (3.378, 64.789)	3
Upright posture as inducement	Yes/No	“with upright posture” = 0, “without upright posture” = 1	<0.001	8.465 (2.654, 26.992)	2
QTd	31 ms	“QTd <31 ms” = 1, “QTd ≥ 31 ms” = 0	0.011	4.263 (1.394, 13.041)	1

In the second part of the study, an external validation test was performed. Predictive values of the scoring model were shown in Table 3. The negative and positive predictive values of the scoring model were 78.8 and 91.7%, respectively, and the accuracy was 80.8%.

**Table 3:** Predictive values of scoring model in external validation.

**Prediction score**	**Clinical diagnosis**	
	**PPS (*n* = 25)**	**VVS (*n* = 53)**
≥3	11	1
<3	14	52

*PPS, psychogenic pseudosyncope; VVS, vasovagal syncope*.

## Discussion

Accurate and timely diagnosis is critical to the follow-up treatment of VVS and PPS patients, and clinical differential diagnosis between these two diseases can be challenging. Therefore, an easy and efficient method for disease differentiation is needed. In this study, we explored the clinical features of VVS and PPS patients and established a useful multivariate predictive logistic regression model to facilitate differential diagnosis. A scoring model was also constructed to simplify the clinical decision, which showed fair agreement with the logistic regression model. Both models revealed a high efficiency in the differential diagnosis between the two diseases and would simplify and improve current clinical practices.

It is difficult to differentiate VVS from PPS for several reasons. Firstly, PPS episodes may resemble VVS, as both involve apparent loss of consciousness, accompanied by similar prodrome symptoms (4). Previous studies have reported distinct traits of PPS in the clinical setting (8, 15). Tannemaat et al. found that eye closure during the onset and long durations of TLOC were more common in patients with PPS compared to those with VVS (8). However, direct clinical observation alone is not sufficient for accurate diagnosis. Direct observation of syncopal attacks rarely occur in clinics. Secondly, although HUTT is useful for distinguishing VVS from PPS (3, 16), it requires special facilities inaccessible in most elementary hospitals. The application of HUTT is also limited due to the risks of inducing severe complications (10).

However, the pathophysiology of VVS and PPS is distinct. VVS results from imposed orthostatic pressure followed by instant circulatory compensation by virtue of the sympathetic and parasympathetic nervous system for proper BP and HR regulation (17). By contrast, the pathogenesis of PPS is not associated with the autonomic nervous system disturbance (13). QTd is an index measured in a 12-lead ECG, which was first proposed by Day et al. (18). It represents the myocardial inconsistency of ventricular repolarization caused by inhomogeneous distribution of autonomic nerves in the ventricle and the duration of heterogeneity of myocardial cell action potentials (19, 20). It has been suggested that patients with augmented ventricular repolarization heterogeneity, measured by elevated QTd, are predisposed to VVS (21). According to previous findings, diabetic autonomic neuropathy may be related to abnormal QTd (22). It was also proposed that populations with autonomic dysfunction are more susceptible to arrhythmias associated with prolonged QTd values (18). Therefore, fluctuations in QTd might be associated with the changes in autonomic nervous function (22), suggesting QTd as a potentially promising indicator for differential diagnosis between the two diseases.

In our study, we initially assessed the clinical characteristics of VVS and PPS. There was no statistical significance in age, gender and BMI. According to the results, patients with VVS were more likely to have experienced standing upright before the onset of apparent TLOC. Typical VVS usually had precipitating triggers such as orthostatic stress (6), which was in accordance with our study. Orthostatic stress gives rise to over-excitation of the adrenergic nervous system, which then leads to excessive ventricular myocardium contraction and activation of vasomotor centers. Subsequently, the Bezold-Jarisch reflex is triggered by the excitation of vagal activity, characterized as peripheral vasoconstriction and hypovolemia, resulting in hypoperfusion of the brain and syncope (23). However, there were no significant differences between patients with VVS and patients with PPS in emotional stress, which was another common precipitating factor in VVS. We speculated that episodes of patients with PPS might also be triggered with psychological stresses, such as anxiety, depression or life events (3), which might be mixed up with emotional stress. Furthermore, the duration of the apparent TLOC was shorter in VVS patients than in PPS patients. Interestingly, most of the patients with VVS regained consciousness within 1–2 min after the onset of syncope, whereas for PPS, the attack time lasted up to approximately 50 min in some cases. This distinction was consistent with that observed in another large-scale retrospective study (24).

Baseline SBP, DBP, HR, and QTd were measured in our study. According to previous reports, baseline DBP and HR were significantly lower in the VVS group than in the control group (25), which might be correlated with a deficient circulating volume in VVS patients (26). The relevance between over-excitation of vagal activity and the decreases in baseline HR and BP have also been proposed (27). In our study, baseline HR was significantly different between VVS and PPS cases, in accordance with previous studies (8). In our study, we likewise found a significant difference between the two diseases based on QTd values. This suggests the potential value of QTd in differentiating VVS and PPS.

This was the first study to develop logistic regression and scoring models to facilitate the differential diagnosis between those two diseases in children. According to the first part of our study, the scoring model has relatively good sensitivity and fair specificity. The negative and positive predictive values of the scoring model in our validation test were 78.8 and 91.7%, respectively, and the accuracy was 80.8%. In our study subjects, we have determined that some PPS patients were characterized by a relatively short syncopal duration, which led to an underestimated percentage of the diagnosis. Further exploration of the above findings should be performed in the future. Overall, the outcome of efficiency in predicting PPS is superior to most situations, considering the widespread overlook and poor identification of PPS in clinical practice (16).

Conclusively, we found that upright posture, syncope duration, family history of syncope, supine HR, and QTd differed significantly between VVS and PPS patients. The present two-centered study significantly improved the differential diagnosis between VVS and PPS. It showed that prolonged syncope duration, presence of upright posture and relatively short QTd were independent predictive factors of PPS. The newly established scoring model is featured with an acceptable sensitivity and specificity in differential diagnosis and largely improves the clinical practice. In the scoring system, three points were given for a duration (“D” for short) of syncopal episode > 30 min; two points for those without upright (“U” for short) posture as an inducement factor; and one point for QTd (“Q” for short) < 31 ms, where “DUQ” for easy memory. With the help of the scoring model, physicians in outpatient and elementary hospitals could reliably suggest a preliminary reference for subsequent treatment after diagnosis (28). For example, counter-pressure maneuvers, midodrine hydrochloride, and oral rehydration saline intake are recommended for VVS patients (29, 30), whereas psychological consultation and cognitive behavioral therapy are indicated for PPS patients (12). Patients would benefit from instant treatment if their diseases can be determined.

There were several limitations to the study. Firstly, selection bias may exist because some PPS patients with mild or atypical symptoms were not sent to cardiovascular clinics initially (16). Secondly, the sample size was relatively small. Larger scaled investigations should be carried out in the future.

## Conclusion

The scoring model consisting of three variables is an easy-to-perform, inexpensive, and non-invasive measure in the initial differential diagnosis between VVS and PPS. It would help with further appropriate management for patients after the initial diagnosis. Future studies are required to prove the generalizability of the predictive measure.

## Data Availability Statement

The datasets generated for this study are available on request to the corresponding author.

## Ethics Statement

The studies involving human participants were reviewed and approved by Ethics Committee in Peking University First Hospital, China; and Ethics Committee in Children's Hospital of Fudan University, China. Written informed consent to participate in this study was provided by the participants' legal guardian/next of kin.

## Author Contributions

ZZ, JD, LH, HT, LT, and XJ had full access to all the data in the study and took responsibility for the integrity of the data and the accuracy of the data analysis. ZZ, XJ, SC, LH, HT, LT, and JD contributed conception and design of the study. ZZ, XJ, LH, HT, CT, and LT collected data. ZZ, XJ, SC, JD, and CT performed the statistical analysis and drafted the manuscript with contributions from CT and SC. All authors contributed to manuscript revision led by JD and HT, and read and approved the submitted version.

### Conflict of Interest

The authors declare that the research was conducted in the absence of any commercial or financial relationships that could be construed as a potential conflict of interest.

## Abbreviations

VVS: Vasovagal syncope
PPS: Psychogenic Pseudosyncope
TLOC: transient loss of consciousness
HUTT: head-up tilt test
SBP: systolic blood pressure
DBP: diastolic blood pressure
QTd: QT-dispersion
ROC: operation characteristic curve
AUC: area under curve
BMI: body mass index.
