# Supplementary material for: Differential Diagnostic Models Between Vasovagal Syncope and Psychogenic Pseudosyncope in Children
Source: Front Neurol. 2020 Jan 23;10:1392. doi: 10.3389/fneur.2019.01392 (PMC6989585; doi:10.3389/fneur.2019.01392)
Supplement: Supplementary file 1 [file Table_1.pdf]

Supplementary Material

Table S1 Baseline demographic data of patients diagnosed with VVS or PPS

**Groups**	**Cases (n)**	**Gender (n, M/F)**	**Age (years)**	**Weight (kg)**	**Height (m)**	**BMI (kg/m^2)**
VVS	150	72/78	11.35 (10.93, 11.78)	45.83 (43.37, 48.29)	1.54 (1.51, 1.56)	18.93 (18.32, 19.55)
PPS	26	14/12	10.73 (9.80, 11.66)	44.32 (37.79, 50.86)	1.49 (1.42, 1.55)	19.49 (17.77, 21.20)
χ^2/Z	-	0.303	-1.139	-0.844	-1.424	-0.477
p-value	-	0.58	0.18	0.40	0.15	0.63

BMI, body mass index; F, female; M, male; PPS, psychogenic pseudosyncope; VVS, vasovagal syncope
